# Smoking by Boys

**Published:** 1893-03

**Authors:** 


### SMOKING BY BOYS.

That the essential principle of tobacco, that which gives it all its value to the smoker, is a virulent poison, is universally admitted. It is agreed also that its primary effect is upon the brain and spinal cord, with a paralyzing tendency.

Even those who defend the moderate use of tobacco admit that its effects are disastrous on some classes of persons. It withers some while fattening others ; causes in some dyspepsia and constipation, while with others it has a contrary effect. It is soothing to some, but induces in others all the horrors of extreme nervousness. Among the brain working class of our population the proportion of those who can use tobacco with impunity is yearly diminishing, as a nervous tendency more and more prevails among us.

Now, whatever may be urged in favor of moderate smoking later in life, all intelligent persons who have given the subject attention unite in condemning the use of tobacco by the young.

Young persons do not know whether or not they belong to the class most liable to be injured by tobacco. No one denies the danger of its excessive use, and the young have neither the intelligence nor the self-control to resist the tendency of smoking to grow into an uncontrollable habit. Further, the brain and nervous system of youth are specially susceptible to the baneful influence of the poisonous principle of tobacco.

That commanding medical authority, the London *Lancet*, says : It is time that the attention of all responsible persons should be seriously directed to the prevalence and increase of tobacco smoking among boys. Stunted growth, impaired digestion, palpitation and other evidences of nerve exhaustion and irritability have again and again impressed the lesson of abstinence, which has hitherto been far too little regarded.

It cites a case which lately came before the coroner for Liverpool,—death from a fatty change in the heart due mainly to smoking cigarettes and cigar ends,—and adds: This, of course, is an extreme example. It is, however, only a strongly colored illustration of effects on health which are daily realized in thousands of instances. Not even in manhood is the pipe or cigar invariably safe. Much less can it be so regarded when it ministers to the unbounded whims and cravings of heedless urchins.

